# Antioxidant intervention of smoking-induced lung tumor in mice by vitamin E and quercetin

**DOI:** 10.1186/1471-2407-8-383

**Published:** 2008-12-20

**Authors:** Jie Yang, Lu Wang, Zhaoli Chen, Zhi-Qiang Shen, Min Jin, Xin-Wei Wang, Yufei Zheng, Zhi-Gang Qiu, Jing-feng Wang, Jun-Wen Li

**Affiliations:** 1 Chinese Center for Disease Control and Prevention, Beijing 100050, PR China; 2 Institute of Environment and Health, No.1, Dali Road, Tianjin, 300050, PR China; 3 School of Public Health, Jilin University, Changchun 130021, PR China

## Abstract

**Background:**

Epidemiological and in vitro studies suggest that antioxidants such as quercetin and vitamin E (VE) can prevent lung tumor caused by smoking; however, there is limited evidence from animal studies.

**Methods:**

In the present study, Swiss mouse was used to examine the potential of quercetin and VE for prevention lung tumor induced by smoking.

**Results:**

Our results suggest that the incidence of lung tumor and tumor multiplicity were 43.5% and 1.00 ± 0.29 in smoking group; Quercetin has limited effects on lung tumor prevention in this in vivo model, as measured by assays for free radical scavenging, reduction of smoke-induced DNA damage and inhibition of apoptosis. On the other hand, vitamin E drastically decreased the incidence of lung tumor and tumor multiplicity which were 17.0% and 0.32 ± 0.16, respectively (p < 0.05); and demonstrated prominent antioxidant effects, reduction of DNA damage and decreased cell apoptosis (p < 0.05). Combined treatment with quercetin and VE in this animal model did not demonstrate any effect greater than that due to vitamin E alone. In addition, gender differences in the occurrence of smoke induced-lung tumor and antioxidant intervention were also observed.

**Conclusion:**

We conclude that VE might prevent lung tumor induced by smoking in Swiss mice.

## Background

Smoking, including second-hand smoking, poses a serious threat to public health. Smoke induced-lung tumor has become one of the malignancies with highest incidence and mortality worldwide [1-3]. Therefore, increasing attention has been paid to searching for effective approaches to the prevention and treatment of smoke-induced lung tumor, including the use of antioxidants. Although the Beta-Carotene Tumor Prevention Study Group demonstrated that beta-carotene may actually promote the development of lung tumor in male smokers rather than prevent the disease [4], the majority of epidemiological findings support the preventive effects of antioxidants, including quercetin and vitamin E (VE), on smoke-induced lung tumor [5-11]. Moreover, in vitro studies have shown that quercetin and VE can prevent the proliferation, invasion, and metastasis of tumor cells and promote the apoptosis of lung tumor cells [12-20]. However, to our knowledge, no animal studies have addressed the potential of quercetin and VE for intervening in the development of smoke-induced lung tumor.

In the present study, we attempted to test the hypothesis that quercetin and VE can prevent lung tumor induced by tobacco smoke (TS) using an intervention model of smoke-induced lung tumor in Swiss mice [21-23]. Surprisingly, although epidemiological and in vitro studies suggested that quercetin has preventive effects on lung tumor, no in vivo preventive effects were observed in this model of smoke-induced lung tumor. However, the present study demonstrated that VE does exert preventive effects in the same animal model. These results may be helpful in future studies aimed at the chemoprevention of TS-induced lung tumor.

## Materials and methods

### Animals

Approximately 5-week-old male and female Swiss mice weighing 18–20 g were purchased from the Laboratory Animal Center of the Academy of Military Medical Science (Beijing, China); 25 mice were housed per cage under standardized conditions with free access to pelleted food or test diet and tap water. All studies were performed with the approval of the Experimental Animal Committee at our Institute.

### Determination of the effects of antioxidant intervention on TS-induced mouse lung tumor

The environmental tobacco smoke system used in the present study was described previously [21,23,24]. Briefly, mice were exposed to a mixture of 89% sidestream and 11% mainstream smoke generated from burning the cigarettes. Chamber atmospheres were monitored for nicotine, CO, and total suspended particulates (TSP). Within the exposure chambers, all cages were periodically rotated so that each cage occupied at least once all possible locations within the exposure chambers. After an initial acclimatization period of 5 weeks involving increasing concentrations of tobacco smoke within the chamber, tobacco smoke concentrations used for the individual experiments ranged from 50 and 150 mg/m^3 ^of TSP. The nicotine, CO, and TSP concentrations for the individual experiments are listed [see additional file 1].

Four hundred and fifty mice were randomly divided into 7 groups: control (C) group and tobacco smoke exposure (TS) group (n = 100, both sexes in each group); quercetin control (C+Q) group, VE (alpha-tocopherol) control (C+VE) group, tobacco smoke plus quercetin (TS + Q) group, tobacco smoke plus VE (TS + VE) group, tobacco smoke plus quercetin and VE (TS + Q + VE) group (n = 50, both sexes in each group). The mice were exposed to 6 h of smoke each day, 5 d a week for 5 months, followed by a 4 month recovery period. The VE and quercetin dose was 100 or 80 mg/kg mouse/d., respectively [25]. The amount of vitamin E and quercetin in the control was also 100 or 80 mg/kg mouse/d. VE and quercetin (reduced) both were purchased from Sigma, St. Louis, MO, USA. The tobacco brand used in this experiment was Hongmei cigarettes (produced by Yuxi Hongta Group, Yunnan, China; purchased from supermarket; tar content, 15 mg and nicotine content, 1.2 mg).

In this study, the quercetin and VE-containing diet was given to the mice, and the daily intake of these antioxidants was calculated strictly by body weight and adjusted weekly. In order to provide an accurate estimate of quercetin and VE lost during dietary processing, HPLC was employed to measure the amount of these two antioxidants in the foods.

### Determination of the oxidation and antioxidation indices in mouse serum

The activities of superoxide dismutase (SOD), glutathione peroxidase (GSH-Px), and the levels of malondialdehyde (MDA) and VE in each group (n = 12, both sexes in each group) were measured using xanthine oxidase method, 5,5'-Dithiobis(2-nitrobenzoic acid) colorimetric method, thiobarbituric acid method and phenanthroline colorimetry, respectively. Colorimetric determination of hydroxyl free radical from Fenton reaction was carried out to represent the levels of reactive oxygen species (ROS). All these corresponding commercial kits were purchased from Nanjing Jiancheng Bioengineering Institute, Nanjing, China. The protocol was carried out according to the manufacturer's instructions.

### Evaluation of tumor formation and pathology

Toward the end of the experiments (nine months), the mice were anesthetized and peripheral blood was collected using orbital sinus venipuncture. To analyze the incidence and multiplicity of lung tumor, the lung of each mouse was harvested and half the sample was fixed in Tellyesniczky's solution (70% ethanol, formaldehyde, and acetic acid in a ratio of 20:5:3) [26], and the other half was snap-frozen in liquid nitrogen. The fixed partial lung lobe was embedded in paraffin, sectioned, and HE stained for pathological analysis. The incidence and multiplicity (the average number of tumor in the lung surface) were expressed in terms of the results of the following equations:

Incidence = tumor cases in each group/total number of surviving mice in the same group × 100

Multiplicity = total multiplicity in each group/total number of surviving mice in the same group

### Single cell gel electrophoresis (SCGE) assay

The SCGE assay (or comet assay) was performed as described by Singh et al. with minor modifications [27]. Briefly, lung tissues were collected immediately when mice were sacrificed at the end of the experiments (nine months) from different groups (n = 20, both sexes in each group) and cut to small pieces. Cells were harvested by trypsinizing and combined with floating cells in the medium containing soybean trypsin inhibitor (Invitrogen). The cells were pelleted and resuspended with cold PBS at 10^5 ^cells/ml. A 5 μl murine lung cells mixed with 75 μl of 0.6% low melting point agarose was transferred to a microscope slide precoated with 1.0% normal melting point agarose and covered with a coverglass for 2 min. When the top layer of agarose was solidified, the coverglass was carefully removed and the slides were submerged into analysis solution to remove cellular proteins. Electrophoresis was then carried out for 20 min. at 25 V, 300 mA. After electrophoresis, the slides were neutralized with Tris-HCl (0.4 M, pH 7.5) and stained with ethidium bromide (0.05 mM). DNA migration was viewed by fluorescence microscopy.

The comet cell tail length (the distance from the center of the comet head to the end of comet tail) in the images was measured using a public domain PC-image analysis program CASP software, and used as the index of the degree of DNA damage.

### Analysis of cell apoptosis

The fresh lung cells enzyme-digested from the whole lung tissue were prepared as the above methods (n = 20, both sexes in each group). Cells were harvested by trypsinizing and combined with floating cells in the medium containing soybean trypsin inhibitor (Invitrogen). The apoptosis analysis was performed as described by Li et al. [28]. Apoptosis was determined by analysis of the percentages of cells with subdiploid DNA content by propidium iodide staining followed by flow cytometry.

### Statistical analysis

The mean and standard deviation were calculated for each group. A one way analysis of variance (ANOVA) was used for comparison of more than two groups. An unpaired two tailed t-test analysis was performed following the ANOVA, or when only two groups were being compared. For Lung tumor incidence, and for the frequency distribution of the different degrees of DNA damage between the experiment groups in SCGE assay, significance of the difference was determined by chi square test. We used the SPSS 11.5 program for the statistical analysis. A P value of ≤ 0.05 was considered significant.

## Results

### Changes in mouse body weight during and after tobacco smoke exposure

**Figure 1:**
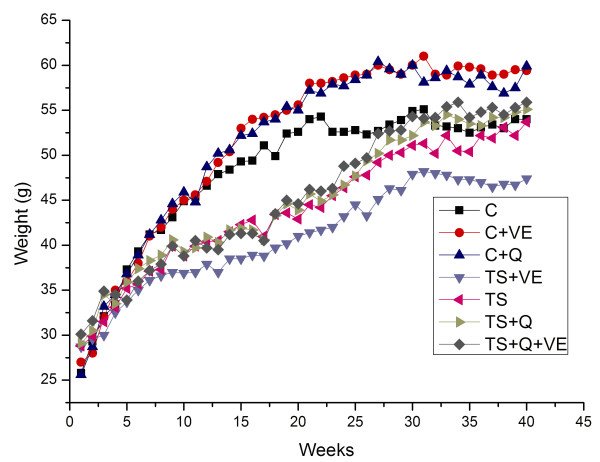
**Changes of body weight in female mice from different groups**. After exposure to TS for 10 to 20 weeks, female mice exhibited much lower body weight gain compared with their counterparts in the three control groups (C, C+VE and C+Q) (p < 0.05), which returned to a basal level after TS withdrawal. The arrow points end of the smoke exposition.

All animals demonstrated good tolerance to tobacco exposure and antioxidant intervention. No death caused by experiment factors was observed throughout the study. In general, body weight gain in the TS exposure group was lower than that in the normal control group (C) and the antioxidant control group (C+Q, C+VE, and C+Q+VE). After tobacco smoke withdrawal, the body weights of TS exposed mice nearly returned to baseline. No significant changes in body weight were observed between the TS-exposed male mice and the male control mice (p > 0.05) [see additional file 2]. After exposure to TS for 10 to 20 weeks, female mice exhibited much lower body weight gain compared with their counterparts in control group (p < 0.05), which returned to a basal level after TS withdrawal (Figure 1).

### There are differences in lung tumor development in mice dependent upon gender and exposure to antioxidants

In the TS exposure group, the incidence of lung tumor and tumor multiplicity were 43.5% and 1.00 ± 0.29, which were significantly higher than those in the normal control group and the C+VE group (p < 0.05); The incidence of lung tumor and tumor multiplicity in VE-treated mice were 17.0% and 0.32 ± 0.16, which were significant lower than those in the TS group (p < 0.05); Quercetin might decreased the incidence of lung tumor and tumor multiplicity compared with TS group, which were 31.1% and 0.76 ± 0.27, respectively, but the difference was not significant (p > 0.05). The tumor preventive effects of the combination of VE and quercetin were not significantly different from that observed when VE was used alone (Table 1).

**Table 1:** Multiplicity and incidence of lung tumor in different groups.

Group	N*	Lung tumor multiplicity			Lung tumor incidence(%)		
			
		Male	Female	Mix	Male	Female	Mix
Control	98	0.20	0.17	0.21 ± 0.08	15.3	6.2	10.7
C+VE	46	0	0.05	0.02 ± 0.01	0	4.8	2.4
C+Q	50	0	0.40	0.14 ± 0.07	0	20.0	12.0
TS	99	1.15^a^	0.80^a^	1.00 ± 0.29^a^	46.5^a^	40.0^a^	43.5^a^
TS+Q	47	1.07	0.36	0.76 ± 0.27	42.9	18.2	31.1
TS+Q+VE	49	0.93	0.13^b^	0.53 ± 0.22	26.7	13.3	21.7
TS+VE	48	0.17^b^	0.44	0.32 ± 0.16^b^	16.7^b^	18.8^b^	17.0^b^

^a^Compared with the Control, C+VE, C+Q, TS+Q+VE, and TS+VE groups, the incidence and tumor multiplicity were significantly increased (p < 0.05); ^b^Compared with TS group and TS+Q group, the incidence and tumor multiplicity were significantly decreased (p < 0.05). (C-control group, VE-Vitamin E treated group, Q-Quercetin treated group, TS-tobacco smoke exposure group).

*N: surviving mice in each group. There were 13 mice died of fighting and infected during the experiments.

Gender differences in tumor incidence and multiplicity were observed in all groups (Table 1). The natural incidence and multiplicity of lung tumor in the male (15.3% and 0.2) were higher than that in female group (6.2% and 0.17), (p > 0.05). The incidence and multiplicity of lung tumor in the VE and/or quercetin control male group was 0, and that in female groups were 4.8%, 20%, and 0.05, 0.4, respectively. The incidence of lung tumor and multiplicity in male mice exposed to TS were 46.5% and 1.15, which were higher than those in females, but not to a significant level (p > 0.05). VE administration significantly decreased the incidence and multiplicity in both genders compared with TS group (p < 0.05). The incidence and multiplicity of lung tumor in quercetin-treated groups (both genders) were slightly lower than those in their counterparts in TS groups (p > 0.05). The incidence of lung tumor and tumor multiplicity in male mice treated with quercetin were remarkably higher than those in their female counterparts (p < 0.05). Differences in the incidence of lung tumor and tumor multiplicity in VE-treated mice were also observed, but were higher in females than in males. However, this difference was not significant (p > 0.05). In the combination treatment group, the tumor incidence and tumor multiplicity in male mice were significantly higher than in females (p < 0.05 and p < 0.01; Table 1).

### Detection of pathology

Histopathology revealed that all the lung tumor nodules were solid lung adenomas. These adenomas comprised well-circumscribed areas of proliferating polygonal-to-cuboidal cells. The proliferating cells lined and filled the alveoli, occasionally exhibiting cytoplasmic vacuolization and mucus production. Foci showing hyperchromasia and cytologic and/or nuclear atypia were observed in some adenomas. The normal pulmonary architecture was destroyed, and the adjacent parenchyma was compressed by the lung tumor nodules.

### Changes in antioxidant status in mouse serum

Serum ROS activity in the TS group was significantly higher than the three control groups (C, C+Q, and C+VE) and the VE-treated ( TS+VE ) group (p < 0.05, Table 2). The ROS activity in the quercetin-treated group (TS+Q) was also much higher than the three control groups and the TS+VE group (p < 0.05, Table 2). The ROS activities in the TS+VE group and the combination treatment group were much lower than the other groups (p < 0.05, Table 2). These results suggest that the TS exposure induces the production of ROS, which can be scavenged by VE, but not quercetin.

**Table 2:** VE concentration, ROS activity, SOD activity, GSH activity and MDA content in mice serum from different groups (mean ± sd, n = 12)

Group	Vitamin E (μg/ml)	ROS (U/ml)	SOD (U/ml)	MDA (mmol/ml)	GSH-Px (U/ml)
Control	6.05 ± 1.81	1002.66 ± 112.72	164.79 ± 32.49	9.84 ± 1.16	746.03 ± 10.19
C+VE	14.23 ± 2.88^b^	511.23 ± 85.45	200.87 ± 30.22	9.22 ± 1.24	800.94 ± 12.55
C+Q	5.01 ± 0.82	811.04 ± 43.54	152.27 ± 26.45	10.28 ± 1.12	752.16 ± 11.68
TS	3.82 ± 2.55^a^	1238.71 ± 33.63^c^	131.93 ± 29.49^e^	10.54 ± 1.20	679.66 ± 6.25^f^
TS+Q	7.11 ± 1.74	1155.87 ± 111.85^d^	157.11 ± 16.96	9.95 ± 1.59	689.33 ± 4.32^f^
TS+ VE	12.08 ± 0.88^b^	577.88 ± 92.47	213.01 ± 25.35	9.97 ± 1.13	677.36 ± 4.50^f^
TS+VE+Q	12.02 ± 1.33^b^	748.43 ± 43.36	226.05 ± 33.14	9.76 ± 0.96	667.56 ± 8.10

^a^VE was significant lower than that in other groups (p < 0.05); ^b ^VE in VE treated groups was significantly higher than those in other control groups (p < 0.05). ^c^ROS activity was significantly higher than that in control groups and VE treated groups; ^d^ROS activity was significantly higher than that in C+Q, C+VE, TS+VE and TS+VE+Q groups. ^e^The level of SOD activity was significantly lower than that in VE treated groups. No statistical significance was observed on MDA content in different groups.^f^GSH-Px activities were significantly lower than those in control groups.

The serum SOD levels in the TS exposure group were the lowest among all the groups. The serum SOD activity in TS+Q group increased slightly, but did not reach a statistically significant level (p > 0.05). The serum SOD levels in each of the VE-treated groups (C+VE, TS+VE, and TS+VE+Q) were significantly higher than in the TS (untreated) group (p < 0.05). There were little or no differences in the serum MDA levels between the different groups (p > 0.05). The GSH-Px activities in the three control groups were much higher than in the treated groups (p < 0.05; Table 2).

The serum VE levels are also presented in Table 2. For the TS exposure group, the level was much lower than in the control groups (p < 0.05). As expected, the serum VE levels in VE-treated groups were much higher than other groups (p < 0.05), and the serum VE levels in quercetin-treated groups were essentially the same as in the control groups The serum VE level in the combination treatment group (TS+ VE +Q) was essentially the same as that in TS+VE group (p < 0.05).

There were no gender differences in SOD, GPx, MAD, ROS and VE levels in different groups.

### VE, but not quercetin, decreases TS-induced DNA damage in mouse lung cells

**Figure 2:**
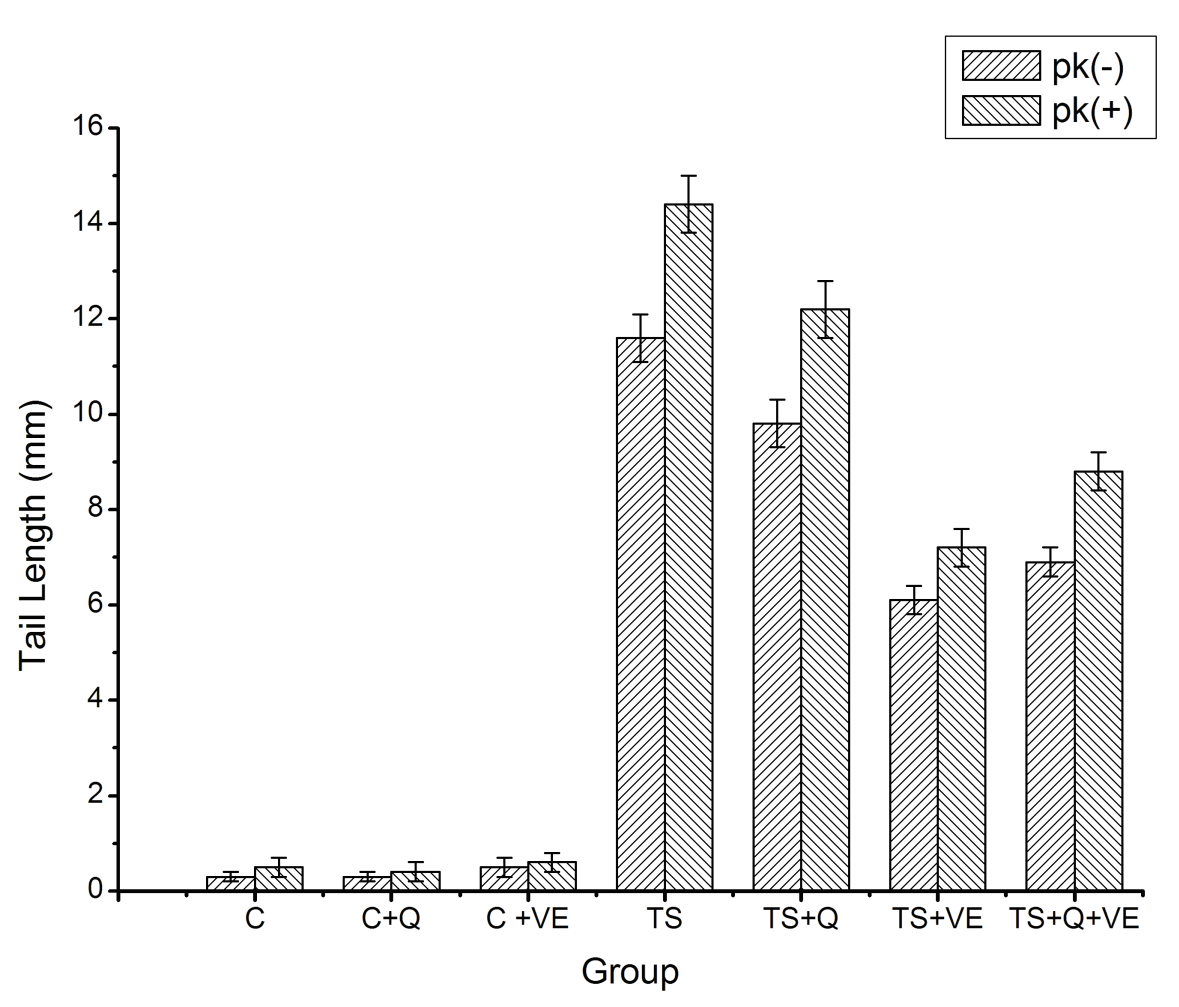
**DNA damage in lung cells from different groups (comet assay)**. The length of the DNA comet tail in the TS group was longer than that from the three control groups and the TS+VE and TS+Q+VE groups (p < 0.05); the length of DNA after digestion with proteinase K [PK(+)] in all TS groups increased significantly compared with non-digested samples [PK(-)].

The length of the DNA comet tail in mouse lung cells exposed to TS was not only longer than in the three control groups (p < 0.05), but was also higher than that in VE-treated and combination treatment groups (p < 0.05). However, no significant differences were observed between the TS and quercetin-treated groups (p > 0.05). After being digested with proteinase K, the travel distance of DNA in all TS exposure groups (TS, TS+Q, TS+VE, and TS+Q+VE) increased significantly compared with non-digested samples, and the extent of the increase in distance was much higher than for control groups (p < 0.05), suggesting that TS exposure induces crosslinking damage to DNA and proteins in mouse lung cells, and that the VE treatment prevents the TS-induced DNA damage (Figure 2).

### Effects of antioxidants on TS-induced apoptosis in mouse lung cells

**Figure 3:**
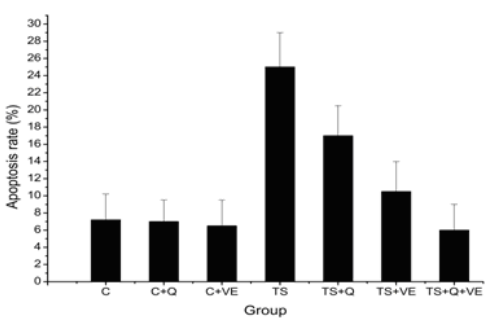
**Comparison of the apoptosis rate of lung cells in different groups**. The rate of apoptosis in the TS and TS+Q groups were significantly higher than the control group (p < 0.05). When compared with the TS group, the apoptosis rates in the TS+VE and TS+Q+VE groups were significantly lower (p < 0.05).

Apoptosis was more prevalent in the group exposed to TS compared to all of the control groups (p < 0.05). VE treatment significantly decreased the TS-induced apoptosis (p < 0.05). This effect was also observed in mice receiving the combined treatment (p < 0.05). However, the quercetin treatment did not result in significant changes in apoptosis compared to the TS group (p > 0.05) (Figure 3). The findings suggest that TS promotes apoptosis in mouse lung cells, which can be reversed by VE treatment, but not quercetin.

## Discussion

The present study represents our efforts to determine the tumor preventive effects of quercetin and VE on TS-induced lung tumor in vivo. Based on an animal model of TS exposure previously described by others [21-24,29], we established a Swiss mouse model of smoke-induced lung tumor in order to evaluate the effects of the antioxidants quercetin and VE. Our findings indicate that the incidence of lung tumor and the tumor multiplicity in the TS exposure group were significantly elevated, suggesting that TS is a potent lung carcinogen for mice. The incidence and tumor multiplicity in quercetin-treated mice were close to those of the TS group, suggesting that quercetin does not have any significant preventive effects under these experimental settings. In terms of the influence of quercetin on serum redox state, DNA damage and cell apoptosis, we also found that quercetin did not exhibit any significant preventive effects. Although many have reported its involvement in antioxidation [9], reduction of DNA damage [12-15] and promotion of tumor cell apoptosis [16-20] in in vitro studies, the in vivo chemopreventive effects of quercetin are uncertain.

Current observations in the literature suggest that vitamin E may be a suitable candidate for the adjuvant treatment of cancer [30]. Our results confirm that VE can significantly reduce the incidence of lung tumor and tumor multiplicity in vivo, to a much greater extent than that was observed with the quercetin. Prevention of TS-induced lung tumor in mice by VE is probably associated with its antioxidant action and effects on reduction of DNA damage and lung cell apoptosis [9]. Other reports indicate that VE was effective inhibitor of cell cycle progression (arrest in G0/G1 phase)[31], and that gamma-Tocopherol and mixed vitamin E forms induced cell death by interrupting the de novo sphingolipid pathway in a prostate cancer cell line[32]. In our current study, we also demonstrated that TS significantly increased ROS activity and reduced the SOD level in mouse serum, which were partially reversed by intervention with VE. This proposed mechanism of action correlates with the incidences of lung tumor in both groups, suggesting that the generation of ROS and the reduced level of SOD in the serum are involved in the lung carcinogenesis. Supplementation with VE increased the serum SOD level and effectively scavenged harmful free radicals in mice.

Experimental studies in animals and in vitro have suggested DNA damage is an important factor in carcinogenesis [33-35]. Vitamin E are sufficient to prevent DNA damage [36,37]. TS can cause DNA damage in mouse lung cells by facilitating the formation of chemical cross-links between DNA and proteins, an event that can be prevented by VE supplementation in this study.

In addition, cigarette smoke can result in Oxidative damage, apoptosis and lung injury, apoptosis may play a crucial role in cigarette smoke-induced lung damage [38]. Our results also indicate that TS can significantly increase apoptosis in lung cells. Apoptosis may be a mechanism by which unwanted cells were eliminated and gene mutation is prevented. But in our study lung cell apoptosis can be counteracted by VE supplementation. Other reports indicate that cell apoptosis was attenuated by antioxidation of VE [39,40]. The mechanism of apoptosis inhibition probably is related with reduction of DNA damage by VE. These advantages offered by VE may combine to reduce the incidence of TS-induced lung tumor in mice.

In summary, we found that TS could induce lung tumor in mice, and that quercetin did not exhibit significant preventive effects under the current experimental conditions. Moreover, no antioxidant effects or reduction in DNA damage induced by TS, or inhibition of TS-induced apoptosis in vivo were observed in quercetin-treated mice. In contrast, VE significantly decreased the carcinogenic potential of TS, and significantly reduced TS-induced DNA damage and cell apoptosis. The combination of VE and quercetin did not show any obvious synergistic or additive effects compared with VE alone. Significant gender differences were observed in TS-induced lung tumor and antioxidant intervention in mice. Further studies are needed in order to unravel the mechanisms by which TS induces mouse lung tumor, and to determine why gender differences were observed in the response to intervention with VE, as well as the relationship between VE dosage and prevention of TS-induced lung tumor in mice.

## Abbreviations

VE: vitamin E; TS: tobacco smoke; TSP: total suspended particulates; SOD: superoxide dismutase; GSH-Px: glutathione peroxidase; MDA: malondialdehyde; ROS: reactive oxygen species; SCGE: Single cell gel electrophoresis.

## Competing interests

The authors declare that they have no competing interests.

## Authors' contributions

JY designed and performed the experiments, and contributed to manuscript writing. LW, ZC, ZS and MJ performed the Single cell gel electrophoresis and apoptosis assay experiments. X-WW, YZ, Z-GQ, JW performed the animal experiments. J-WL designed the experiments and wrote the manuscript. LW and ZC also evaluated tumor formation and pathology.

## Pre-publication history

The pre-publication history for this paper can be accessed here:



## Supplementary Material

Additional file 1 **Exposure Data for Carcinogenesis Assay with different group Mice.** Exposure parameter for Carcinogenesis Assay with different group Mice, such as Relative humidity, Temperature, CO, TSP, and Nicotine. Click here for file

Additional file 2 **Changes of body weight in male mice from different groups.** There is no significant different in the changes of body weight in male mice from different groups (p > 0.05). The arrow point shows smoking exposure is stopped. Click here for file
