# Supplementary material for: Antioxidant intervention of smoking-induced lung tumor in mice by vitamin E and quercetin
Source: BMC Cancer. 2008 Dec 20;8:383. doi: 10.1186/1471-2407-8-383 (PMC2625366; doi:10.1186/1471-2407-8-383)
Supplement: Additional file 1 — Exposure Data for Carcinogenesis Assay with different group Mice. Exposure parameter for Carcinogenesis Assay with different group Mice, such as Relative humidity, Temperature, CO, TSP, and Nicotine. [file 1471-2407-8-383-S1.doc]

Table 1 Exposure Data for Carcinogenesis Assay with different group Mice

| Parameter | Weeks 1-2 | Weeks 3-5 | Weeks 6-20 |
| --- | --- | --- | --- |
| Relative humidity (%) | 70.13±12.04(8) | 60.67±6.42(15) | 66.00±4.82(75) |
| Temperature (℃) | 23.56±1.12(8) | 21.83±1.32(15) | 22.48±0.99(75) |
| CO (ppm) | 168.75±29.00(8) | 213.33±32.40(15) | 256.52±34.98(75) |
| TSP (mg/m^3^) | 75.37±11.67(8) | 107.44±16.96(15) | 119.32±28.12(75) |
| Nicotine (mg/m^3^) | 8.32±2.12(8) | 10.05±4.87(15) | 12.55±6.08(75) |

Note. All measurements were made within the inhalation chambers. Data are given as mean±SD; Number of determinations is in parentheses. CO, carbon monoxide; TSP, total suspended particulates.
