# Supplementary figures and images for: Antioxidant intervention of smoking-induced lung tumor in mice by vitamin E and quercetin
Source: BMC Cancer. 2008 Dec 20;8:383. doi: 10.1186/1471-2407-8-383 (PMC2625366; doi:10.1186/1471-2407-8-383)

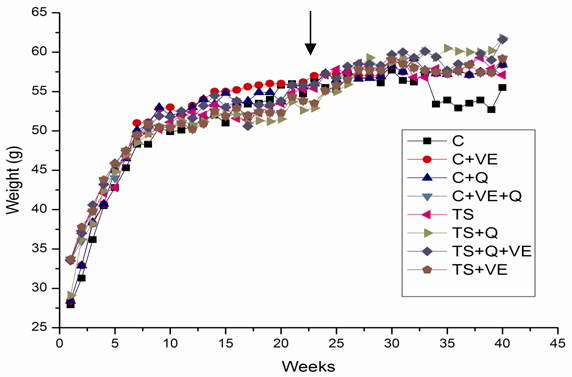

Supplement: Additional file 2 — Changes of body weight in male mice from different groups. There is no significant different in the changes of body weight in male mice from different groups (p > 0.05). The arrow point shows smoking exposure is stopped. [file 1471-2407-8-383-S2.tiff]
